# Advanced airway management in hoist and longline operations in mountain HEMS – considerations in austere environments: a narrative review This review is endorsed by the International Commission for Mountain Emergency Medicine (ICAR MEDCOM)

**DOI:** 10.1186/s13049-018-0490-5

**Published:** 2018-04-03

**Authors:** Urs Pietsch, Jürgen Knapp, Oliver Kreuzer, Ludwig Ney, Giacomo Strapazzon, Volker Lischke, Roland Albrecht, Patrick Phillips, Simon Rauch

**Affiliations:** 1 Department of Anaesthesiology and Intensive Care Medicine, Cantonal Hospital St. Gallen, Rorschacher Strasse 95, 9007 St. Gallen, Switzerland; 2 Air Zermatt, Emergency Medical Service, Heliport Zermatt, 3920 Zermatt, Switzerland; 3 Bergwacht (German Mountain Rescue Service), Carstennstrasse 58, 12205 Berlin, Germany; 4 Department of Anaesthesiology and Pain Medicine, Inselspital, Bern University Hospital, University of Bern, Freiburgstrasse 10, 3010 Bern, Switzerland; 5 Department of Anaesthesiology, University Hospital of Munich (LMU), 80336 Munich, Germany; 6 Institute of Mountain Emergency Medicine, EURAC Research, Viale Druso 1, 39100 Bozen, Italy; 7 Swiss Air-Ambulance (REGA), P.O. Box 1414, 8058 Zurich, Switzerland; 8 Travis County STAR Flight, 7800 Old Manor Rd, Austin, TX 78724 USA

**Keywords:** Helicopter emergency medical services, Search and rescue, Airway management, Hoist, Longline, Human external cargo, Prehospital emergency medicine

## Abstract

**Background:**

Providing sufficient oxygenation and ventilation is of paramount importance for the survival of emergency patients. Therefore, advanced airway management is one of the core tasks for every rescue team. Endotracheal intubation is the gold standard to secure the airway in the prehospital setting. This review aims to highlight special considerations for advanced airway management preceding human external cargo (HEC) evacuations.

**Methods:**

We systematically searched MEDLINE, EMBASE, and PubMed in August 2017 for articles on airway management and ventilation in patients before hoist or longline operation in HEMS. Relevant reference lists were hand-searched.

**Results:**

Three articles with regard to advanced airway management and five articles concerning the epidemiology of advanced airway management in hoist or longline rescue missions were included. We found one case report regarding ventilation during hoist operations.

The exact incidence of advanced airway management before evacuation of a patient by HEC is unknown but seems to be very low (< 5%). There are several hazards which can impede mechanical ventilation of patients during HEC extractions: loss of equipment, hyperventilation, inability to ventilate and consequent hypoxia, as well as inadequacy of monitoring.

**Conclusions:**

Advanced airway management prior to HEC operation is rarely performed. If intubation before helicopter hoist operations (HHO) and human cargo sling (HCS) extraction is considered by the rescue team, a risk/benefit analysis should be performed and a clear standard operating procedure (SOP) should be defined. Continuous and rigorous training including the whole crew is required. An international registry on airway management during HEC extraction would be desirable.

## Background

Survival of severely injured patients is time dependent. It is known that the use of a helicopter emergency medical service (HEMS) can significantly shorten rescue times, especially in mountainous areas [1], and can improve patient outcomes [2].

Due to the difficult terrain in the mountains, landing a helicopter is not always possible, and hovering and human external cargo (HEC) operations such as helicopter hoist operations (HHO) or longline/human cargo sling (HCS) operations are utilized. A hoist is a mechanical system in which a steel cable attached to the center of the helicopter’s underside is lowered and a patient is hoisted up to the helicopter in either a harness, a rescue bag or a litter. A HCS is a fixed length of line, with a hook to which rescuers and patients can be attached [3].

In a multi-center retrospective study, Tomazin et al. found that 6 of 9 HEMS bases in four European countries had the capability to rescue casualties by HEC operation from difficult and remote geographical and topographical locations [3]. Yet data from the 6121 rescue missions analyzed in their study did not differentiate between simple (landing the helicopter) and difficult (utilizing HEC) technical rescue procedures.

Guidelines for medical interventions, especially for airway management in casualties that require evacuation by HEC operation, are limited. The medical commission of the International Commission for Alpine Rescue (ICAR), the leading organization for mountain rescue medicine, does not provide clear and up-to-date recommendations.

Between 70% and 90% of the patients rescued in HEC operations in Europe suffer from traumatic injuries sustained in mountain regions where difficult terrain precludes helicopter landings and injuries typically involve recreational activities [1, 4, 5]. In the winter Alpine environment, most of the patients treated in HEC operations have had ski and snowboard accidents, whereas in the summer months hiking and climbing accidents are predominant. Most victims are male and have only minor injuries [6]. They commonly need intravenous drug therapy (especially for pain), as well as wound treatment and splinting of fractured bones or distorted joints.

There are some severely injured patients, however, who need advanced airway management before evacuation from the site of an accident by hoist or longline. Thus, the aim of this position paper is to describe the epidemiology of advanced airway management in HEC operations and to provide evidence-based practical advice on indication and performance.

## Material and methods

A literature search was performed as a text word search in order to include articles not yet indexed with MeSH terms. Using the search terms “airway management AND hoist”, “airway management AND (aircraft OR helicopter) AND winch”, “airway management AND HEMS”, “epidemiology AND advanced airway management AND (hoist OR longline)” we searched MEDLINE, EMBASE and PUBMED in August 2017. Additionally, the authors reviewed the reference lists of the articles retrieved by the electronic searches to find other relevant reports not indexed in the electronic databases.

The authors independently screened the titles and abstracts of all articles identified by the search. Fifty-three peer-reviewed articles were eligible for inclusion. Full text was obtained for all selected papers and analyzed by two authors (UP, JK) independently with regard to the topic of our review.

## Results

From a total of 53 citations identified, three were found to be relevant with regard to advanced airway management in HEC operations (Table 1). Seven articles were found reporting characteristic of patients and medical interventions with possible advanced airway management in HEC operations (Table 2). We found only one case report regarding ventilation during a hoist operation [7].

**Table 1:** Literature of advanced airway management in hoist or longline rescue missions

Study	Study design	Setting	Patients	Findings
Lavon O [7]	Case study	Hoist rescue missions with mechanical ventilation of the patient using the Oxylator EM-100®	intubated prehospital trauma patients (ISS > 15), *n* = 5	Automated flow-limited mechanical ventilation is efficient for ventilating adult patients with protected airway during the short periods of hoist rescue and facilitates a smooth rescue operation.
Burns BJ [8]	Case study	One intubated prehospital trauma patient underwent resuscitation bag ventilation during a stretcher hoist. Manikin model of bag ventilation during a stretcher hoist in downwash of an AW 139 helicopter, with two resuscitation bags with differing compliances.	intubated prehospital trauma patient, *n* = 1 manikin model, *n* = 2	50 ft under the rotor disc, the resuscitation bag failed due to compression by the downwash. The stiffer, less compliant resuscitation bag did not fail in the manikin model.
Murphy D [9]	Randomized crossover trial	Adults suspended in single sling, double sling, supine in a rescue stretcher, and in a rescue basket. Primary variables measured were FEV 1, FVC, FEV 1/FVC ratio, and IC in each modality versus control. Secondary measurements: peripheral oxygen saturation, heart rate, and respiratory rate.	healthy adults, spontaneous breathing, *n* = 27	The rescue basket was not associated with any change in measured outcomes. The stretcher was associated with small decreases in expiratory volumes, but an increase in IC. Single sling had detrimental effects on respiratory function (not applicable after advanced airway management).

*FEV*-1 forced expiratory volume in one second, *FVC* forced vital capacity, *IC* inspiratory capacity, *ISS* injury severity score

**Table 2:** Characteristics of patients and medical interventions in human external cargo missions

Study	Study design	Setting	Patients	Findings
Corniche J [10]	Prospective study. Review of primary rescue interventions in all missions involving hoisting of a physician, Switzerland, 1998 to 2002	1855 HEMS missions, 156 (8%) HEC missions with physician, 133 patients (7%) for analysis, pre-alpine region of Switzerland	trauma: *n* = 102 (77%) minor trauma: *n* = 14 (10%).	medical interventions: • major analgesia with sedation: *n* = 4 (3%) • fracture reduction: *n* = 5 (4%) • intravenous fluid administration (> 1500 ml): n = 4 (3%) • endotracheal intubation: *n* = 5 (4%), (point of time not mentioned)
Sherren PB [11]	Prospective study. Review of physician only interventions in all missions involving hoisting of a physician, Australia, 2009 to 2012	1582 HEMS missions, 130 (8%) HEC missions with physician, 120 patients (8%) for analysis, remote and inaccessible regions of New South Wales, Australia	trauma: *n* = 108 (90%)	63 physician only interventions in *n* = 48 (40%) patients: • advanced analgesia: 44 (70%) • advanced airway management: 5 (8%) (point of time not mentioned) • circulatory support: 3 (5%) • orthopedic manipulation of joints/limbs: 6 (10%) • thoracostomy: 1 (2%) • diagnostic ultrasound: 1 (2%) • hypertonic saline administration: 3 (5%)
Ausserer J [12]	Retrospective registry study. Review of trauma patients with the aim to identify victims sustaining major trauma during recreational activities in mountainous terrain, Austria 2011 to 2013	58 major trauma victims (Injury Severity Score ≥ 16), 40 (69%) HEC operations, in remote and mountainous areas in the State of Tyrol	head/neck trauma: *n* = 25 (35%) chest trauma: *n* = 27 (37%)	medical interventions: • ATLS before HEC operation: *n* = 30 (75%) • advanced airway management: *n* = 23 (40%) (point of time not mentioned)
Ney L [6]	Retrospective study. Air Zermatt database, Switzerland, 2010 to 2015	10,027 HEMS missions, 2808 (28%) HEC evacuations, in the high alpine regions of Switzerland		HEC evacuation and NACA-Score ≥ 4: *n* = 602 (21% of all HEC evacuations)
Ruppert M [21]	Retrospective registry study. Review of all primarily admitted patients with and without hoist rescue, Germany, 2006 to 2015	20,241 HEMS missions, 1813 (9%) HEC operations, two HEMS bases in the pre-alpine regions of Germany	trauma in HEC missions: *n* = 850 (71%) major trauma in HEC missions: *n* = 127 (11%) cardiovascular diseases in HEC missions: *n* = 129 (11%)	medical interventions in HEC missions: • reposition of joint or limb: *n* = 190 (11%) • endotracheal intubation: *n* = 83 (5%) (point of time not mentioned) • CPR: *n* = 41 (2%) • thoracostomy: *n* = 8 (0.4%) • intraosseous access: *n* = 6 (0.3%)
Pasquier M [4]	Retrospective study. Review of medical interventions in all missions involving hoisting of a physician, Switzerland, 2003 to 2008	9879 HEMS missions, 921 (9%) HEC operations, in the alpine region of Switzerland	trauma in HEC operations: *n* = 840 (91%) paediatric patients (≤15 years): *n* = 56 (6%)	HEC operation and NACA-Score ≥ 4: *n* = 246 (27% of all HEC operations) medical interventions in HEC missions: • major analgesia: *n* = 478 (52%) • vasoactive drugs: *n* = 26 (2%) • endotracheal intubation: *n* = 16 (2%) (at the scene of accident) • intravenous fluid administration (> 1000 ml): *n* = 16 (2%) • CPR: *n* = 7 (1%)
Carpenter J [22]	Retrospective study. Review of patient demographics hoisted in the backcountry of Utah, USA, from 2001 to 2011	171 HEC missions with 214 patients	trauma in HEC missions: *n* = 146 (68%)	Medical interventions in HEC missions: • CPR: *n* = 5 (2%)

*ATLS* advanced trauma life support, *CPR* cardiopulmonary resuscitation, *HEC* human external cargo, *HEMS* helicopter emergency medical service, *NACA* National Advisory Committee for Aeronautics

Lavon et al. [7] demonstrated that ventilation with an automated flow-limited mechanical ventilation device, like the Oxylator EM-100®, is efficient for ventilating adult patients with protected airways during the short periods of hoist rescue (*n* = 5). There were no alterations in the parameters of endtidal CO_2_, oxygen saturation, and pulse rate from the beginning of the automated mechanical ventilation with the Oxylator until the conversion back to bag-valve ventilation. Additionally, the authors state that this device facilitates a smoother rescue operation due to the fact that bag-valve ventilation is not necessary and the rescuer can concentrate on the completion of a safe HEC operation. However, it should be taken into account that the Oxylator offers no visual monitoring of correct ventilation and the acoustic feedback is inaudible during HEC operation.

In a case report, Burns et al. [8] reported on one intubated prehospital trauma patient undergoing bag-valve ventilation during a litter hoist. Fifty feet under the rotor disc, the resuscitation bag failed. After that, they studied bag-valve ventilation during a litter winch in the downwash of an AW 139 helicopter in a manikin model with two types of resuscitation bags with different compliances. Whereas ventilation failed with the more compliant resuscitation bag due to compression by the downwash, the stiffer, less compliant resuscitation bag was not compressed and allowed adequate ventilation.

Murphy et al. [9] performed a randomized crossover trial studying the influence of different positions and rescue devices (rescue basket, litter, single sling) during hoist or longline rescue on the respiratory function in 27 healthy spontaneously breathing adults. They found that the rescue basket was not associated with any change in measured respiratory outcome parameters. The litter was associated with small decreases in expiratory volumes, but an increase in inspiratory capacity, whereas single sling evacuations had detrimental effects on respiratory function.

### Epidemiology of advanced airway management in HEC missions

Frequency and procedures of HEC operations differ significantly with different HEMS providers. Corniche et al. [10] reported 156 helicopter hoist operations (HHO) out of a total of 1855 helicopter rescues in their mixed (urban/rural/alpine) operational area around Lausanne (Switzerland) over a four-year period, indicating a frequency of 8.4%. Seventy-seven percent of the patients in this study rescued by HEC operations had traumatic injuries. Nine (5.8%) casualties requiring HHO had an injury severity score (ISS) > 15 and 5 (3.2%) of them needed endotracheal intubation prior to hoist extraction. Four of them had a traumatic injury, and one a severe facial injury.

Sherren et al. [11] reported 130 HHO out of 1582 helicopter rescue missions of the Greater Sydney Area HEMS (GSA-HEMS) from August 2009 to January 2012, which corresponds to a frequency of HHO of about 8%. Consistent with the numbers from the Swiss study, four casualties (3.1%) had to be intubated prior to HHO, and in one patient a surgical airway was required [11].

Air Zermatt/Switzerland performed 842 HEC operations for severely injured patients (NACA ≥3) between January 2010 and September 2016 in the rural and high-Alpine area of southwestern Switzerland. Endotracheal intubation had to be performed in 19 patients (2%) before HEC removal. In contrast, the International Alpine Trauma Registry, collecting data on severely traumatized patients (ISS > 15) exclusively in the Alpine regions of South Tyrol (Italy) and Tyrol (Austria), reported a need for endotracheal intubation prior to HEC evacuation in 5 of 40 patients (13%) [12].

In summary all studies listed in Table 2 report a relevant number of patients in HEC operations with the need for advanced airway management at some point of time during the rescue mission, which points out the need for recommendations on advanced airway management in HEC operations.

## Discussion

Evacuation of an intubated casualty using HEC is a very rare and highly complex scenario. Due to the case study character and the small numbers of patients in the existing literature, our literature review cannot generate evidence, but we can provide some informative recommendations on advanced airway management in HEC operations.

### Medical and tactical considerations in an austere environment

1. Be careful: mountains can be unforgiving. Victims and rescuers face extreme temperatures, strong winds with high wind chill factors, risk of avalanches and falling rocks, and a persistent danger of falls from heights or into crevasses. Therefore, immediate evacuation out of the extreme environment is often required. Medical tactics are dictated by those factors, and benefits and risks of medical interventions need to be carefully weighed. There is no gold standard established for austere rescue environments, as every mission is a unique combination of factors. Critical thinking on the part of the entire team is paramount.
2. Train continuously and interprofessionally: During Alpine and other complex rescue missions, the HEMS medical provider and other rescue personnel may not know each other before deployment. These teams will organize themselves in an “ad hoc” fashion after the operation has already commenced. Hence, there is an increasing need for these teams to undergo joint and structured training that not only includes technical skills, but also non-technical skills, such as communication, situational awareness, decision making, as well as stress and resource management.
3. Follow the guidelines: Every effort possible and reasonable under the harsh environmental conditions should be made to provide state-of-the-art and up-to-date emergency medicine according to the current guidelines: Advanced Cardiac Life Support (ACLS), Prehospital Trauma Life Support (PHTLS), Advanced Trauma Life Support (ATLS), European Trauma Course (ETC), Pediatric Advanced Life Support (PALS), Tactical Combat Casualty Care (TCCC).
4. Avoid the 4 H’s: Hypoxia, hypothermia, hypoglycemia, and hypovolemia need to be avoided at all times during the evacuation.
5. Collect data to improve: There are no up-to-date recommendations from the International Commission for Mountain Emergency Medicine (ICAR MEDCOM) [1, 13] or other mountain rescue organizations. Nor do the American College of Surgeons (responsible for ATLS guidelines), the European Resuscitation Council (ERC), the National Association of Emergency Technicians (responsible for PHTLS guidelines) or ICAR MEDCOM define clear recommendations for the treatment of seriously injured or ill patients in the austere or mountain environment. ICAR MEDCOM has published two recommendations regarding airway management in the field and medical standards for mountain rescue operations using helicopters, but they are based on weak evidence and are outdated [1, 13]. Given the low numbers of mountain trauma victims compared to urban trauma victims, collecting international data from multiple mountain HEMS organizations (like the International Alpine Trauma Registry does for Tyrol and south Tyrol) would be beneficial to generate sufficient patient data.

### Indication for advanced airway management prior to human external load extraction

Bag-valve-mask (BVM) ventilation, which requires maintenance of an adequate mask seal, is not feasible during HEC operations. However, endotracheal intubation in exposed terrain and under harsh environmental conditions, as well as the need for manual or mechanical ventilation during HEC extraction after advanced airway management, is associated with difficulties and hazards (Table 3). Therefore, the threshold for advanced airway management prior to extraction should probably be higher than is practiced by ground emergency medical services.

**Table 3:** Difficulties, challenges and possible hazards of rescuing an intubated patient with a HEC operation

Medical difficulties and challenges
Airway device displacement
Hyper−/hypoventilation
Disconnection between airway device and resuscitation bag or respirator
Limited monitoring possibilities (no acoustical and limited visual observation) during HEC operation
Inability to perform any airway device corrections of false placement during HEC operation
Complex and time-consuming securing of all medical devices (e.g., oxygen bottles, monitors, respirator etc.) for HEC operation
Risk of hypothermia due to prolonged exposure to the elements
Non-technical difficulties and challenges, human factors
Limited situational awareness
Increased work load during highly complex rescue maneuvers
Involuntarily shift of focus from safety during hoist operations to medical care
Prolonged exposure to physical hazards (e.g., rockfall) due to prolonged time on scene
Technical difficulties and challenges
Loss of the resuscitation bag or other medical devices, for example due to downwash or gravitational forces
Displacement of the airway device following exposure to downwash, which could interfere with re-inflation of the BVM during manual ventilation

Possible indications for assisted or controlled ventilation during a HEC operation are listed in Table 4.

**Table 4:** Absolute and relative indications for advanced airway management

Absolute indication
Apnea or agonal respiration
Traumatic brain injury (TBI) with insufficient ventilation or oxygenation despite high flow O_2_
Severe (chest) trauma patient with insufficient ventilation or oxygenation despite high flow O_2_
Cardiopulmonary resuscitation (CPR) with mechanical chest compression devices, e.g., due to a hypothermic arrest (after an avalanche accident, a fall into a crevasse, or extreme physical exhaustion) [23]
Relative indication
GCS < 9 with preserved oxygenation

### Emergency anesthesia and analgosedation

The indication for emergency anesthesia and endotracheal intubation has to be evaluated very critically. Besides the terrain, environmental conditions, the factor of time (prolonged time on scene, therefore increased risk of hypothermia), and the patient’s condition, factors related to the medical provider (in particular training level and expertise) also have to be taken into account. Before or during technical rescues, emergency anesthesia should be avoided if possible. Nevertheless, for humanitarian reasons analgosedation of the severely injured patient is indicated. For an adult patient (75 kg body weight), the application of 1–3 mg midazolam and 25 mg esketamine or 50 mg ketamine (if necessary, repetitive administration of 10 mg esketamine or 20 mg ketamine after 10 min) is a practicable initial approach [14].

Rapid sequence induction (RSI) of emergency anesthesia should follow a simple and standardized protocol. Lyon et al. suggest RSI with fentanyl (3 μg/kg body weight), ketamine (2 mg/kg) and rocuronium (1 mg/kg), the so-called 3:2:1 regimen. Reduced doses of fentanyl (1 μg/kg IV) and ketamine (1 mg/kg IV) were administered in patients with hemodynamic compromise, the 1:1:1 regimen [15]. With these regimens (*n* = 145) they report excellent first-pass intubation success (100%) and a hemodynamically stable induction of anesthesia (only one patient with systolic blood pressure < 90 mmHg). For patients “in extremis”, administering a muscle relaxant only to facilitate endotracheal intubation is an option [15].

If hypovolemia is expected, fluid resuscitation is recommended prior to induction of anesthesia, taking into account that patient and environmental conditions often don’t allow for an adequate period of fluid resuscitation. If hypotension occurs [16] after the induction of anesthesia, cardiovascular support with catecholamines (e.g., boluses of 10 mcg norepinephrine or 100 mcg phenylephrine) may be necessary. Therefore, preparation of labelled syringes with circulation stabilizing drugs before RSI in the suspected hypovolemic patient is recommended.

If the decision is made to induce emergency anesthesia in a patient with a suspected pneumothorax, or if there are signs of a pneumothorax after endotracheal intubation in patients prior to hoist or long-line maneuvers, a thoracostomy or chest drain placement has to be performed before evacuation of the patient, as a tension pneumothorax caused by positive pressure ventilation would cause cardiocirculatory collapse within minutes.

### Ventilation during HEC operations

After advanced airway management and during HEC operations, the patient can be ventilated either with a self-inflating bag or with a mechanical ventilator. Given the complete lack of evidence, neither method can be favored. However, in our practice we usually opt for the self-inflating bag, which is fixed to the rescue bag with a string and carabiner. It is light-weight, easy to handle, and the experienced provider gets good tactile feedback about adequate ventilation. Testing the sufficient compliance of the rescue bags for ventilation under real-life conditions during HEC operations is an indispensable prerequisite. It should be taken into consideration that manual ventilation during HEC operation is prone to hypo- or hyperventilation. Especially in patients with traumatic brain injury (TBI) this might have adverse effects.

In contrast, a mechanical ventilator is much heavier and the visual and acoustic feedback is often not discernible due to the noise level and the light reflecting on the monitor. An inadvertent disconnection with possible fatal consequences can thus easily be missed. Some HEMS services use a small and light-weight ventilator such as an Oxylator EM-100®, with a fixed flow and working pressure which requires no power source [17]. However, these devices need pressurized oxygen, which again increases weight and makes handling more complex. Moreover, these ventilators have no monitor and give no visual or acoustic feedback about the quality of ventilation, and a failure during HEC operation might go undetected.

### Education and training

There are no standardized guidelines or trainings established for ALS in the mountain rescue environment, but there are some related courses which can provide helpful information. Among these are the general courses based on the specialty rescue module of the UIAA/ICAR/ISMM Diploma in Mountain Medicine, the WMS Advanced Wilderness Life Support® course, and dedicated courses for airway management in difficult and remote geographical and topographical locations such as the one organized by the medical commission of the Corpo Nazionale Soccorso Alpino e Speleologico (CNSAS, Italian Mountain Rescue Organization), as well as simulations in mountain weather chambers [18].

If intubation is unavoidable before HOC extraction, we suggest a careful risk/benefit analysis following a clear and predefined SOP [19, 20]. Such an SOP could be used in any Search and Rescue (SAR)/HEMS program that carries out hoist and longline maneuvers. Pit stop-like training should be drilled periodically. We encourage these organizations to establish continuous and interprofessional simulation training, with a strong emphasis on a safe procedure of advanced airway management and ventilation, as well as HEC extraction of the ventilated patient. Strong emphasis should be placed on securing the endotracheal tube and securing the resuscitation bag with a cord (i.e., to the rescuer or rescue bag). An accidental dislocation of the endotracheal tube or loss of the rescue bag during HEC extraction cannot be corrected in time and the patient may suffer from hypoxemia for several minutes. Without continuous training and awareness of possible weak points, HEC extraction of an intubated patient is extremely dangerous. Regular training involving the entire team should be undertaken. In our opinion, repeated education – for example, a video-guided debriefing of the simulation training – is a valuable tool for increasing familiarity with HEC procedures.

Tactical considerations due to harsh environmental conditions have to be considered during training. It is of paramount importance that every clinician working in austere or mountain rescue missions is aware of the difference between in-hospital standards and performing pragmatic but still high-quality medicine adapted to the ambient conditions.

## Conclusions

The placement of an advanced airway device prior to HEC extraction is rarely performed. A careful risk/benefit analysis should be undertaken before advanced airway management is performed prior to HEC operation, and a clear SOP should be followed. Equipment for ventilation of patients during HEC operations has to meet specific requirements (e.g., dimensions, fall protection, functionality in strong downwash) and monitoring of correct ventilation is extremely limited. Therefore, continuous pit stop-like training including the whole crew is a valuable tool to achieve a high level of competence. Given the low number of mountain trauma patients, establishing a uniform data-collection method and collecting data in an international database would be beneficial to generate a feasible number of patient data and to gather more evidence to improve the safety of HEC operations.

## Abbreviations

ACLS: Advanced cardiac life support
ALS: Advanced life support
ATLS: Advanced trauma life support
BLS: Basic life support
BVM: Bag-valve-mask
CNSAS: Corpo nazionale soccorso alpino e speleologico
CPR: Cardiopulmonary resuscitation
ETC: European trauma course
FEV-1: Forced expiratory volume in 1 s
FVC: Forced vital capacity
HCS: Human cargo sling
HEC: Human external cargo
HEMS: Helicopter emergency medical service
HHO: Helicopter hoist operation
IC: Inspiratory capacity
ICAR MEDCOM: Medical commission of the international commission for alpine rescue
ICAR: International commission for alpine rescue
ISMM: International society for mountain medicine
ISS: Injury severity score
NACA: National advisory committee for aeronautics
PALS: Pediatric advanced life support
PHTLS: Prehospital trauma life support
RSI: Rapid sequence induction
SAR: Search and rescue
SOP: Standard operating procedure
TBI: Traumatic brain injury
TCCC: Tactical combat casualty care
UIAA: Union Internationale des associations d’alpinisme

## References

[1] Tomazin I, Ellerton J, Reisten O (2011). Medical standards for mountain rescue operations using helicopters: official consensus recommendations of the International Commission for Mountain Emergency Medicine (ICAR MEDCOM). High Alt Med Biol.

[2] Andruszkow H, Hildebrand F, Lefering R (2014). Ten years of helicopter emergency medical services in Germany: do we still need the helicopter rescue in multiple traumatised patients?. Injury.

[3] Grissom CK, Thomas F, James B (2006). Medical helicopters in wilderness search and rescue operations. Air Med J..

[4] Pasquier M, Geiser V, De Riedmatten V, Carron PN (2012). Helicopter rescue operations involving winching of an emergency physician. Injury.

[5] Hearns S (2003). The Scottish mountain rescue casualty study. Emerg Med J.

[6] Ney L. Situational Awareness in Alpine Helicopter Emergency Medical Service. https://www.researchgate.net/publication/311517977_Siuational_Awareness_in_Alpine_Helicopter_Emergency_Medical_Service. Accessed 13 Feb 2018.

[7] Lavon O, Hershko D, Barenboim E (2010). The utility of flow-limited automated mechanical ventilation during airborne hoist rescue missions. Am J Emerg Med.

[8] Burns BJ, Edwards K, House T (2012). Bag valve mask failure during HEMS intubated stretcher winch. Air Med J.

[9] Murphy D, Garner A, Bishop R (2011). Respiratory function in hoist rescue: comparing slings, stretcher, and rescue basket. Aviat Space Environ Med.

[10] Corniche J, Pasquier M, Yersin B, Kern C, Schoettker P (2012). Helicopter rescue involving the winching of a physician. Air Med J..

[11] Sherren PB, Hayes-Bradley C, Reid C, Burns B, Habig K (2014). Are physicians required during winch rescue missions in an Australian helicopter emergency medical service?. Emerg Med J.

[12] Ausserer J, Moritz E, Stroehle M, Brugger H, Strapazzon G, Rauch S, Mair P (2017). International alpine trauma registry study group. Physician staffed helicopter emergency medical systems can provide advanced trauma life support in mountainous and remote areas. Injury.

[13] Thomas A, Rammlmair G, Wiget U. ICAR recommendation Nr. 11: Emergency Intubation and Ventilation on the Field. In: Consensus guidelines on mountain emergency medicine and risk reduction. http://www.alpine-rescue.org/ikar-cisa/documents/2011/ikar20111027000798.pdf. Accessed 13 Feb 2018.

[14] Hossfeld B, Bein B, Boettiger BW, Bohn A, Fischer M, Graesner JT, Hinkelbein J, Kill C, Lott C, Popp E, Roessler M, Schaumberg A, Wenzel V, Bernhard M (2016). Recommended practice for out-of-hospital emergency anaesthesia in adults: statement from the out-of-hospital emergency Anaesthesia working Group of the Emergency Medicine Research Group of the German Society of Anaesthesiology and Intensive Care. Eur J Anaesthesiol.

[15] Lyon R, Perkins Z, Chatterjee D, Lockey D, Russell M (2015). Significant modification of traditional rapid sequence induction improves safety and effectiveness of pre-hospital trauma anaesthesia. Crit Care.

[16] Green R, Butler M (2017). Increased mortality in trauma patients who develop post-intubation hypotension. J Trauma Acute Care Surg.

[17] OXYLATOR® Studien und Referenzen. http://inter-rescue.de/wp-content/uploads/OXYLATORStudienundReferenzen.pdf. Accessed 5 Feb 2018.

[18] Pietsch U, Ney L, Kreuzer O, Berner A, Lischke V (2017). Helicopter emergency medical service simulation training in the extreme: simulation-based training in a mountain weather chamber. Air Med J..

[19] Viren N, Naik MD, Susan E, Brien MD (2013). Review article: simulation: a means to address and improve patient safety. Can J Anaesth.

[20] Kerslak D, Oglesby A, Di Rollo N (2015). Tracheal intubation in an urban emergency department in Scotland: a prospective, observational study of 3738 intubations. Resuscitation.

[21] Ruppert M, van Boemmel T, Lefering R, Fiala W, Gäßler M (2017). The spectrum of helicopter hoist rescue missions. A retrospective ten-year analysis of alpine HEMS missions. Notfall Rettungsmed.

[22] Carpenter J, Thomas F (2013). A 10-year analysis of 214 HEMS backcountry hoist rescues. Air Med J..

[23] Pietsch U, Lischke V, Pietsch C, Kopp KH (2014). Mechanical chest compressions in an avalanche victim with cardiac arrest: an option for extreme mountain rescue operations. Wilderness Environ Med.

